# The application of mass spectrometry imaging in traditional Chinese medicine: a review

**DOI:** 10.1186/s13020-022-00586-8

**Published:** 2022-03-05

**Authors:** Lieyan Huang, Lixing Nie, Zhong Dai, Jing Dong, Xiaofei Jia, Xuexin Yang, Lingwen Yao, Shuang-cheng Ma

**Affiliations:** 1 National Institutes for Food and Drug Control, National Medical Products Administration, Beijing, 102629 China; 2 Shimadzu China Innovation Center, Beijing, 100020 China; 3 Waters Corporation, Beijing, 100176 China

**Keywords:** Mass spectrometry imaging, Traditional Chinese medicine, Medicinal plants, Natural compounds, Spatial distribution

## Abstract

Mass spectrometry imaging is a frontier technique which connects classical mass spectrometry with ion imaging. Various types of chemicals could be visualized in their native tissues using mass spectrometry imaging. Up to now, the most commonly applied mass spectrometry imaging techniques are matrix assisted laser desorption ionization mass spectrometry imaging, desorption electrospray ionization mass spectrometry imaging and secondary ion mass spectrometry imaging. This review gives an introduction to the principles, development and applications of commonly applied mass spectrometry imaging techniques, and then illustrates the application of mass spectrometry imaging in the investigation of traditional Chinese medicine. Recently, mass spectrometry imaging has been adopted to explore the spatial distribution of endogenous metabolites in traditional Chinese medicine. Data collected from mass spectrometry imaging can be further utilized to search for marker components of traditional Chinese medicine, discover new compounds from traditional herbs, and differentiate between medicinal plants that are similar in botanical features. Moreover, mass spectrometry imaging also plays a role in revealing the pharmacological and toxicological mechanisms of traditional Chinese medicine.

## Background

Medicinal plants have been regarded as a treasure-house of therapeutic constituents over the centuries. In China, the application of traditional Chinese medicine (TCM) can be dated back to ancient times, showing validated effectiveness in disease treatments [1, 2]. During clinical application, TCM takes action in a multi-compound and multi-target mode. The former makes it difficult to analyze major components in TCM materials containing a numerous number of chemicals, while the latter brings about confusion when revealing the pharmacological and toxicological mechanisms of TCM.

Up to now, different kinds of analytical approaches have been adopted to look into the pharmaceutically important chemicals in TCM materials. In conventional analytical process of TCM components, tedious pretreatment of samples is usually unavoidable. Before the identification of target chemicals, a complex procedure including extraction, isolation and purification should be performed step by step. Then, crude drug extraction or purified chemicals are analyzed with techniques such as liquid chromatography (LC) [3], gas chromatography (GC) [4, 5], gas chromatography coupled with mass spectrometry (GC–MS) [6, 7] and liquid chromatography coupled with mass spectrometry (LC–MS) [8, 9]. These methods are capable of performing accurate analysis with good reproducibility and low limits of detection. However, the objects analyzed by conventional approaches are usually homogenized tissues, leading to in the loss of spatial information of the analytes. On the other hand, analysis of natural compounds from extraction of medicinal plants costs not only time and labor, but also a massive amount of organic solvent. Besides, extraction process under heating may cause decomposition of thermolabile components [10].

To overcome the obstacles mentioned above, several novel analytical protocols have been developed in the past few decades. In 2007, a method for the direct analysis of alkaloid profiling in plant tissues by using matrix-assisted laser desorption ionization time of flight mass spectrometry (MALDI-TOF-MS) was developed. Component profiles of raw and processed *Aconitum carmichaeli* Debx. (Fuzi in Chinese) were obtained without tedious sample pretreatment procedure [11]. Another investigation succeeded to reveal the spatial distribution of natural compounds in *Sinomenium acutum* stems using matrix assisted laser desorption ionization mass spectrometry (MALDI-MS). Relative abundances of metabolites obtained from MALDI-MS profiles were correlated with their localization regions, thus providing insights into the accumulation patterns of plant metabolites [12]. In 2012, the combination of laser microdissection (LMD) and routine analytical techniques was adopted to demonstrate the spatial distribution of natural compounds in plant tissues. Sinomenii Caulis, sourced from the stems of *Sinomenium acutum* (Thunb.) Tehd. et Wils., was microdissected into different parts and then analyzed with liquid chromatography-quadrupole/time of flight-mass spectrometry (LC-QTOF-MS) respectively. Results revealed that different parts of Sinomenii Caulis contained varied alkaloids [13]. Similarly, the accumulation sites of saikosaponins in three *Bupleurum* species were studied using LMD and ultra-high performance liquid chromatography quadrupole/time of flight-mass spectrometry (UHPLC-QTOF-MS) [14]. More recently, mass spectrometry imaging (MSI) has emerged as a promising technique to quickly analyze in situ natural compounds in TCM without complicated sample treatment.

MSI is a label-free analytical method which allows in situ visualization of tremendous number compounds in sample tissues. Over the years, MSI techniques have undergone continuous improvements in ionization methodologies, instrumental conditions and sample preparation protocols [15]. MSI techniques differ from each other by the way how analytes are ionized. The most commonly applied ionization sources of MSI are matrix-assisted laser desorption ionization (MALDI), desorption electrospray ionization (DESI), and secondary ion mass spectrometry (SIMS). Detailed mechanisms of ionization sources could be accessed in the following section.

During its development, MSI has been widely applied in different areas including biological, pharmaceutical and medical research. For instance, MALDI-MSI was adopted to visualize different components in mammal tissues, such as carboxyl-containing metabolites in rat kidney [16], *N*-glycan species in human brain [17], and lipids within lamina propria cells of porcine colon [18]. MSI has also been utilized to illustrate pharmacological mechanisms of drug [19]. Besides, MSI makes it possible to look into diagnosis and prognosis of disease in an innovative way [20, 21]. Data generated from MSI could provide information of metabolic response in tumor tissues, thus enabling a deeper exploration of tumor microenvironment [22, 23]. In the past few years, researchers have attempted to observe the localization of endogenous metabolites in plant tissues using MSI [24]. The progress made in plant science puts forward the investigation of natural compounds in TCM plants using MSI.

MSI analysis of TCM eliminates the tedious and complicated process of sample pretreatment, which to some extent reduces the risk of compound decomposition that may occur during extraction step. After MSI performance, different classes of natural products can be detected simultaneously in a single display [25]. Mass images intuitively reveal that even within the same organ, chemicals might be distributed to restricted parts, providing novel insights into the biosynthetic mechanisms, physiological functions as well as transportation modes of natural compounds in medicinal plants. Aside from organ-specific analysis, there is also plenty of research that visualizes chemical distribution across different organs, supporting a more comprehensive understanding of how major components are distributed throughout the medicinal plants. Apart from the chemicals that has already been recognized as effective bioactive compounds in TCM, unknown products could also be mapped out using the untargeted analysis function of MSI, offering new inspiration for drug discovery [26].

Generally, TCM is applied in the form of combined formula. The administration of TCM formulation takes action in a multi-target way, which makes it difficult to illustrate the pharmacological mechanisms. MSI have been adopted in a few reports to clearly reveal the target organs of TCM natural compounds in the animal body, providing a potential tool to study the pharmacological behaviors of TCM [27, 28]. Furthermore, MSI may be employed to investigate on toxicological effects of TCM components [29].

This review provides the background information of MSI and makes a comparison between the most commonly applied MSI techniques. Then the article illustrates the application of MSI techniques in TCM research by comprehensive review of plenty of detailed cases, proving that MSI is capable of supporting phytochemical, pharmacological and toxicological investigations of TCM. For the analysis of natural products in TCM plants using MSI, a general workflow is summarized in the text. Data generated from MSI can be directly utilized to obtain the spatial information of natural compounds, or be further analyzed with different mathematical methods to achieve various analytical goals. Hopefully, this review will be instructive to explore the potential of MSI in TCM investigations.

## Main text

### Overview of different ionization sources utilized in MSI

SIMS was first reported by Castaing and Soldzian in 1962 [30, 31]. A few decades later, static secondary ion mass spectrometry was developed by Alfred Benninghoven and was found to be very sensitive for the detection, identification and structural investigation of biologically important compounds, including amino acids, peptides and vitamins [32, 33].

**Fig. 1:**
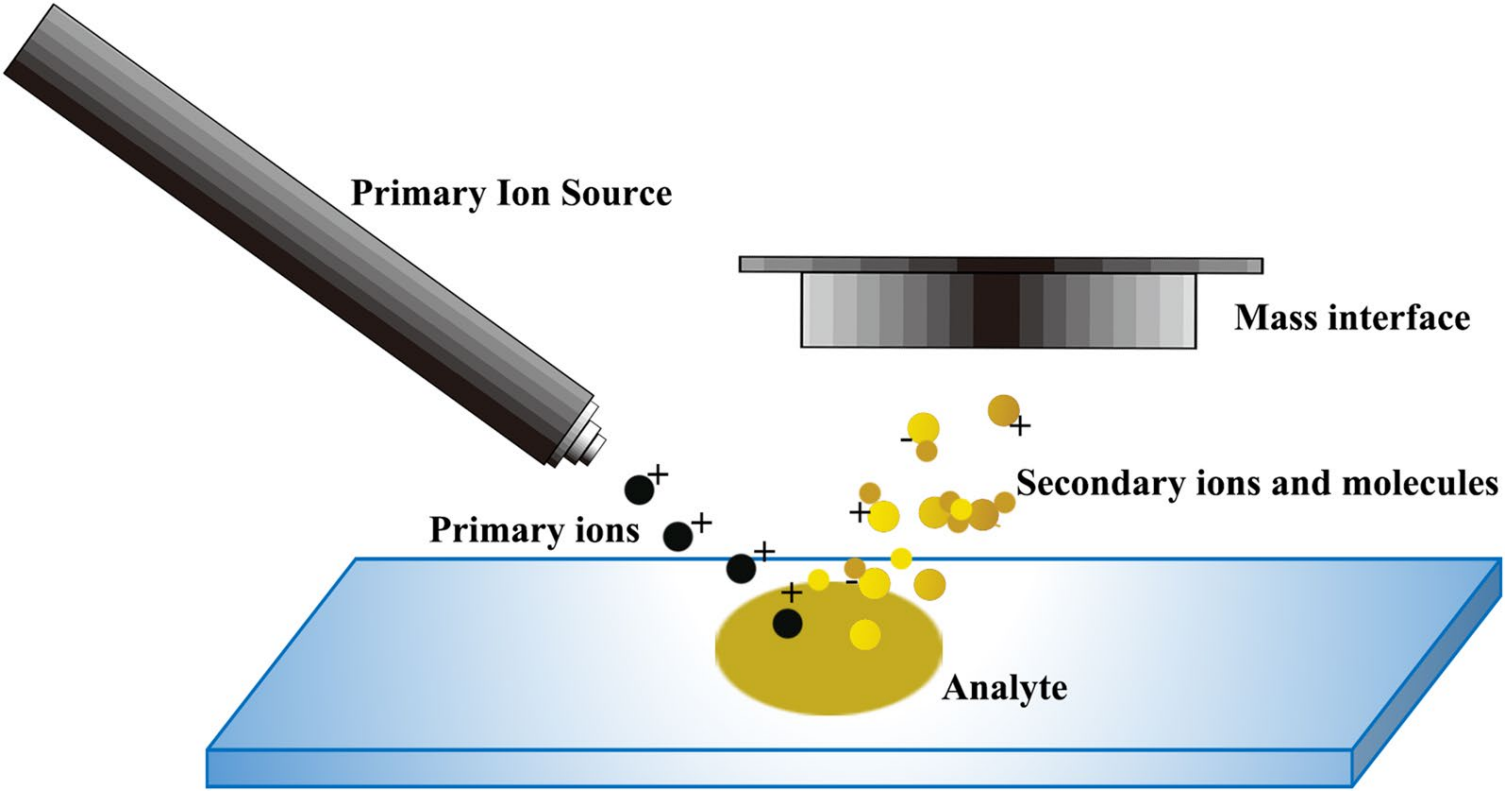
Schematic illustration of the principle of SIMS

During SIMS analysis, secondary ion emission occurs after the impact resulting from a beam of high-energy primary ions focused on the sample surface, then the secondary ions are captured by mass spectrometer to accomplish surface analysis [34]. The schematic illustration of the principle of SIMS could be accessed in Fig. 1. As SIMS develops, ion clusters other than Ga^+^ and In^+^ have been tested as the projectiles hitting the target surface, including Au^3+^, Bi^3+^, SF^5+^, and C^60+^, which provides support for the detection of different analytes [35].

The history of MALDI development could be dated back to 1985 when Karas' group observed that a strong signal of alanine appeared in the mass spectrum of a mixture of alanine and tryptophan, suggesting that tryptophan might serve as an absorbing matrix which enhanced ion yield of the non-absorbing alanine during laser desorption process [36]. In the same year, Koichi Tanaka from Shimadzu Corporation in Japan succeeded in producing gaseous ions of protein molecules using laser pulses, and was awarded The Nobel Prize in Chemistry 2002 for his invention of soft desorption ionization methods for mass spectrometry analysis of biological macromolecules [37]. In Koichi Tanaka's research, a mixture of Cobalt ultrafine metal powder and glycerin was selected as the matrix. Then in 1988, Karas' group achieved the detection of proteins with molecular weight above 10,000 Da using aqueous solution of nicotinic acid as the ultraviolet absorbing matrix [38]. Ever since, a number of compounds were tested as potential matrices, among which the derivatives of cinnamic acid and aromatic carbonyl compounds were commonly applied until now [39].

**Fig. 2:**
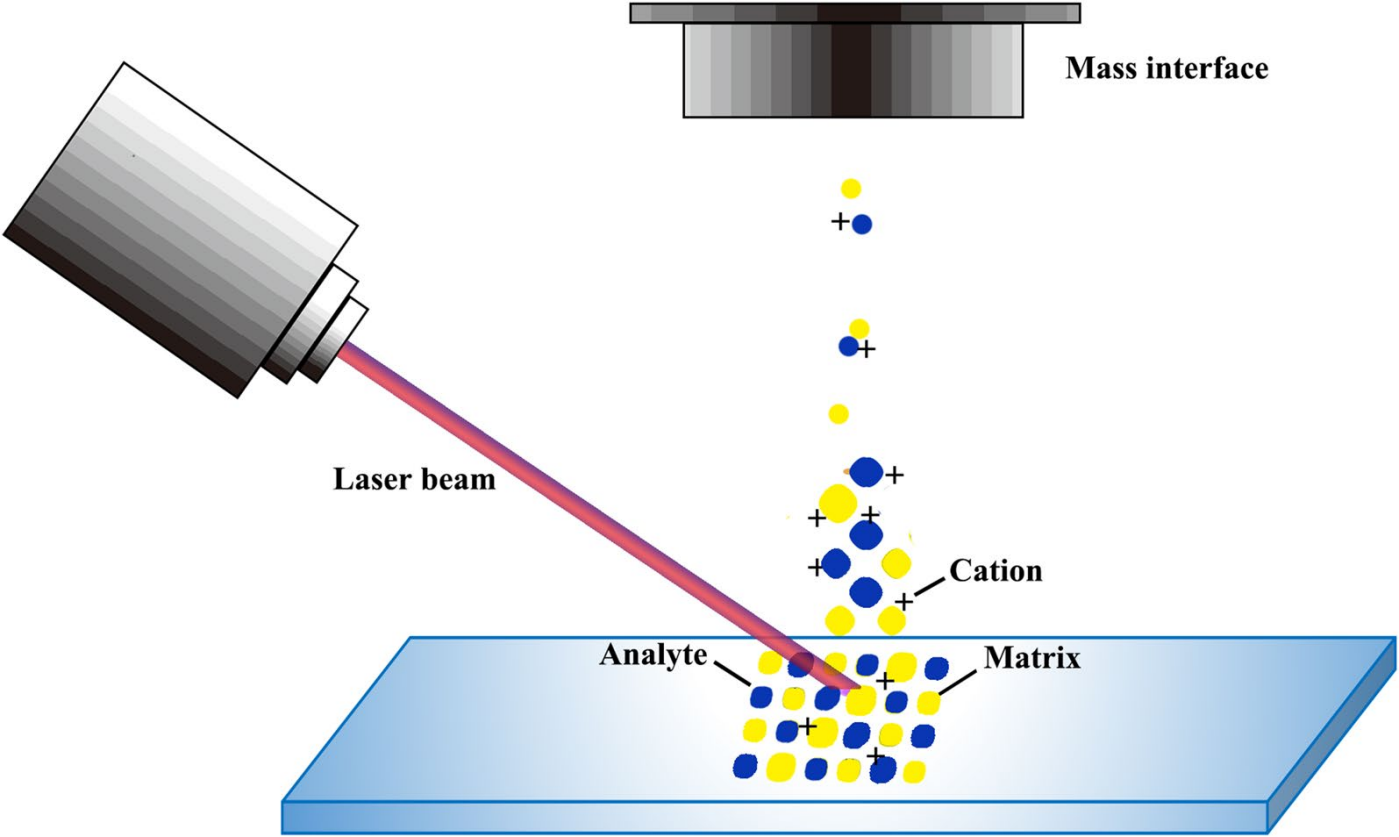
Schematic illustration of the principle of MALDI

Ionization and desorption process in MALDI were investigated for years, many proposed mechanisms were possible [40]. A simple model of MALDI ionization process is displayed in Fig. 2. At the early stage of MALDI development, a photochemical ionization mechanism was applied to explain how molecules were ionized in MALDI analysis [41]. Researchers stated that energy from the irradiation of a pulsed laser was absorbed by matrix molecules, giving rise to the desorption of matrix-analyte complexes. The desorbed analytes then underwent a series of gas-phase reactions, producing protonated/deprotonated or alkali-adducted ions [42]. However, the photochemical ionization mechanism alone was not enough for the explanation of complex procedures that might have occurred in MALDI process. In 2003, the concept for cluster ionization was developed by Karas. In the new theory, the analyte started with a precharged form in the matrix crystals instead of being neutral in the gas phase. When excitational energy density in the matrix reached a critical value, an explosive cluster emission was triggered. Then the precharged analyte ions were separated from their counterions thanks to the mechanical energy supplied by exploding clusters. Finally, ions were formed by the evaporation of neutral species, for example, the matrix molecules. Karas commented that though cluster ionization could be regarded as the dominant ionization process in MALDI, the contribution of photoionization within clusters could not be neglected [43]. Considering the desorption of analytes in MALDI analysis, it was elucidated that the pathways of phase transition had a close relation to the laser parameters and matrix properties [44]. The phase explosion model assumed that transition from condensed phase to gaseous phase occurred when the matrix system became exceedingly unstable during laser pulses, leading to co-desorption of matrix and analytes [45].

**Fig. 3:**
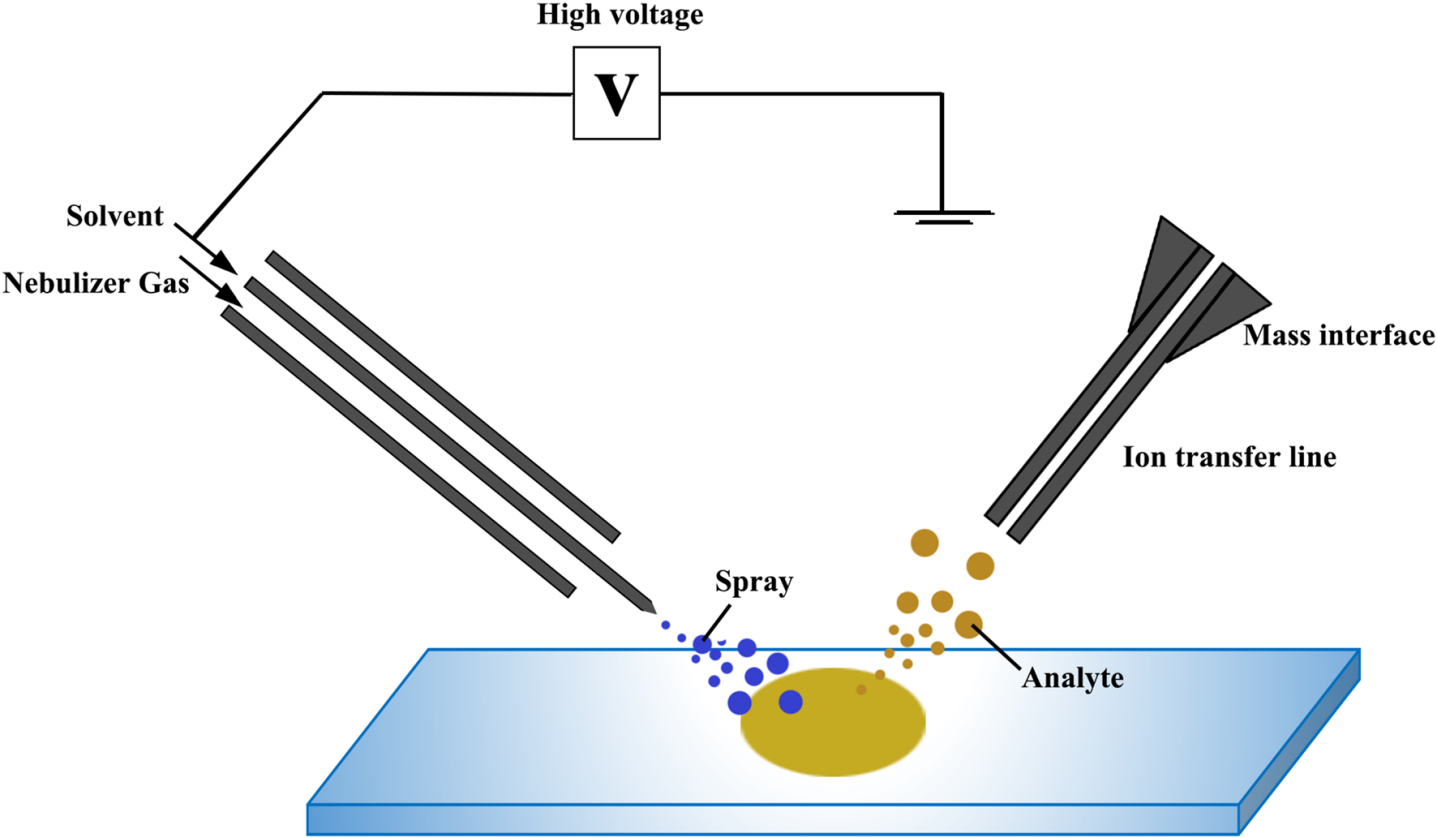
Schematic illustration of the principle of DESI

In 2004, the first report on DESI was published by Graham Cooks' lab at Purdue University. A new method termed DESI was applied to the ionization of a broad range of analytes, including amino acids, alkaloids, terpenoids, peptides and proteins [46]. The DESI ion source consists of an inner capillary responsible for the delivery of spray solvents and an outer capillary responsible for the delivery of nebulizing gas [47], as can be seen from Fig. 3. When charged droplets are directed onto the surface of a sample, the impact of the spray gives rise to gaseous ions of sample molecules. Then the ions are transported along an ion transfer line to reach the vacuum system. Finally, mass spectra of analyzed samples are obtained [48]. During the DESI process, a droplet pick-up method could be adopted to explain the possible mechanism of desorption and ionization of analytes [49]. Charged droplets which collide with sample surface would pick up sample molecules as they splash off the surface, then the secondary droplets containing analytes produce gaseous ions by standard electrospray ionization processes [50, 51].

However, during the ionization and desorption process of DESI, undesirable translocation of sample molecules might arise because of the splashing of spray solvents, which could be overcome by the development of a modified ionization source named nano-DESI. In nano-DESI, a solvent bridge contacting the sample surface is formed between the primary capillary and the nanospray capillary. Afterwards, the solvent containing analytes is transported to a mass spectrometer inlet by self-aspirating nanospray [52]. Since nano-DESI delivers droplets at low velocity, a minimal splashing of spray solvents could be achieved [53].

In the past few decades, the appealing advantages provided by ambient ionization methodologies have attracted many researchers to search for new ambient ionization sources. Basically, ambient ionization techniques can be classified into three main categories according to their desorption mechanisms: liquid extraction, plasma extraction and laser ablation [54]. Commonly applied ambient ionization sources other than DESI are liquid extraction surface analysis (LESA), direct analysis in real-time (DART), and laser ablation electrospray ionization (LAESI). The ionization mechanisms of DESI and LESA are similar, both involving a liquid–solid extraction procedure. DESI utilizes a charged spray of solvents to desorb and ionize analytes, while LESA forms a liquid microjunction between the robotically controlled pipette tip and sample surface to extract analytes from the tissues [55]. In 2005, DART was reported as the first plasma-based ambient ionization source. The DART source exposes a carrier gas, typically nitrogen, helium, or argon to a discharge chamber where a plasma of excited-state species is produced [49]. During DART process, the formation of negative ions has relationship with reactions of oxygen/water cluster ions, while the dominant mechanism of positive-ion formation involves proton transferring [56]. It is believed that the metastable gas molecules are the working reagent in DART [57]. LAESI, however, is one of the laser ablation techniques. In LAESI, a mid-infrared (mid-IR) laser is utilized to ablate a sample surface [54]. Water-containing samples such as fresh plant tissues absorb energy from intense laser pulses, giving rise to the ejection of ablated materials. Next, the ejected molecules are intercepted by an orthogonal electrospray plume. Finally, the ionization process is accomplished [58, 59].

### The development of commonly applied MSI techniques

The potential of SIMS for mass imaging was first reported in 1983 when D. Briggs attempted the development of SIMS for molecular imaging and accomplished the microanalysis of heterogeneous organic surfaces [15]. The imaging mode of SIMS was useful in characterizing biomaterials such as monolayer films, surfaces of particles or powders and delivery systems in drug pellets [60]. SIMS imaging also opened up new possibilities in biological research, for instance, imaging the diverse lipids in cell and tissue samples [61, 62]. Two dimensional ion images collected from serial tissue sections using MSI could be further reconstructed to generate a three dimensional map of analytes within the whole tissue, which is termed as 3D-MSI [63]. In 2007, John S. Fletcher and his colleagues generated the 3D biomolecular images of *Xenopus laevis* oocyte using time of flight-secondary ion mass spectrometry (TOF–SIMS) [64]. To achieve unambiguous identification of biological specimens at high resolution, a new method named TOF–SIMS parallel imaging MS/MS was developed by Gregory L. Fisher [65]. More recently, the 3D OrbiSIMS instrument was invented by Passarelli, which combined the high spatial resolution of SIMS with high mass resolution of an orbitrap, presenting a powerful tool in 3D imaging of biomolecules at subcellular resolution [66].

The first application of MALDI in molecular imaging of peptides and proteins was carried out by Captrioli et al. in 1997, providing mass images of peptides and proteins in biological samples collected from rats and humans [67]. Since then, MALDI-MSI was applied to various areas including cancer research [68], neurobiology [69], and pharmaceutical development [70, 71]. The expansion of MALDI-MSI from two dimensions to three dimensions was initiated by Crecelius et al. in 2005 [72]. A recent investigation visualized lipids in newly fertilized zebrafish embryos using 3D MALDI-MSI, demonstrating the capability of 3D MALDI-MSI to image chemicals within a single cell [73].

Among the three MSI techniques, DESI-MSI is the youngest technique developed by Cooks at the beginning of the twentieth century [74]. Soon after the invention of DESI, this novel ionization source was utilized to investigate on intact biological tissues such as mouse-pancreas, rat-brain, and human-liver adenocarcinoma tissue [75]. Later in 2006, lipids in rat brains were imaged using DESI, and a spatial resolution estimated as better than 500 μm was achieved [47]. In 2010, DESI imaging played a role in cancer diagnostics by discriminating between cancerous and normal tissues of human bladders [76]. Over the years, it has been proved that DESI-MSI is a powerful tool for in situ analysis of biomolecules such as lipids [77] and proteins [78]. Apart from biological samples, various kinds of natural compounds in plant tissues could also be visualized using DESI-MSI [30]. Moreover, based on the knowledge of DESI principles, air flow assisted desorption electrospray ionization (AFADESI) was invented by Z. Abliz's group to support whole-body molecular imaging under ambient conditions [79].

### Comparison between commonly applied MSI techniques

Each of the three MSI techniques has its own weaknesses and strengths depending on the mechanical principles behind. MALDI-MSI, DESI-MSI and SIMS-MSI complement each other in four major aspects: detectable mass range, achievable spatial resolution, sample preparation requirements, and mass performance conditions.

SIMS represents one of the most energetic desorption techniques [80], the practical mass range of which is quite limited owing to the molecular fragmentation resulting from ion impact [42] and the limitation on the size of molecules that can be lifted from a surface [81]. Small molecules up to 2000–3000 Da could be visualized using SIMS imaging [81]. The ambient source, DESI, is also more suitable for the detection of small molecules in the 50–2000 Da range [42]. One of the advantages of MALDI over DESI and SIMS is the ability in offering a broader detectable mass range. For a period of time, MALDI detection of proteins exceeding 25 kDa was limited. Then in 2010, MALDI imaging exceeding 50 kDa was accomplished using a high mass detector [82]. More recently, with the application of a caffeic acid matrix, proteins with high molecular weight close to 200 kDa was successfully imaged by MALDI-MSI [83].

Considering the spatial resolution achievable with each MSI techniques, SIMS is much better than MALDI and DESI since it holds the ability to restrict the analyzed region down to about 100 nm [42]. The introduction of Nano-SIMS led to an even higher lateral resolution at around 50 nm using a cesium primary ion beam [84]. As for MALDI-MSI, the spatial resolution of about 20–30 μm could be provided with commonly applied commercial instruments [85]. In 2015, an optimized strategy of MALDI-MSI was carried out by Caprioli's group, achieving a 1 μm laser spot diameter and a 2.5 μm raster step size [86]. In comparison, the resolution power of DESI-MSI is weaker than MALDI and SIMS due to the electrospray based desorption [42]. Generally, DESI-MSI is capable of providing spatial resolution at 50–200 μm [74]. By changing instrumental parameters such as solvent flow rate, emitter capillary diameter and mass spectrometric scan rate, higher lateral resolution at approximately 35 μm was achieved using DESI-MSI [87].

Prior to the analytical process of MALDI-MSI, matrices should be deposited on the sample surface, usually a spray technique is adopted for matrix application [88]. The co-crystallization between analytes and matrices would influence the ion signal intensities, and the inhomogeneity of formed crystals is one of the problems associated with the poor reproducibility of MALDI profiling [89, 90]. New approaches for matrix application are developed to improve the performance of MALDI-MSI, including matrix coating assisted by an electric field (MCAEF) [91], matrix sublimation, and matrix recrystallization [92]. Another downside of matrix application is that the frequent signals of a selected matrix may interfere with the signals of target molecules [81]. In comparison, both DESI and SIMS can be imaged directly after sectioning without applying matrix, thus not only simplifies the sample preparation before MSI analysis [93] but also avoids the interference of matrix complexes. When dealing with special samples such as the flowers and leaves of plants, direct DESI analysis may be replaced by indirect analysis via imprinting the analytes on sorbent materials such as porous polytetrafluoroethylen (PTFE) or thin layer chromatography (TLC) silica plates [30].

Another difference between the three MSI techniques lies in whether a vacuum circumstance is required during the ionization process. SIMS and MALDI are two ionization sources that must be performed under vacuum circumstances. Consequently, SIMS and MALDI are not suitable for the detection of labile components that are not vacuum stable [42]. Plenty of ambient ion sources have been developed to support MSI performance under atmospheric pressures, including DESI, LESA, DART and LAESI [54, 94]. Besides, the drawback of MALDI gives rise to the invention of a modified MALDI source, namely, the atmospheric pressure matrix assisted laser desorption ionization (AP-MALDI). In AP-MALDI, the ions produced on sample surface are transferred from the atmospheric pressure region to the inlet orifice of the mass spectrometer with the assistance of a stream of nitrogen [95].

### General procedures for MSI analysis of TCM

Based on literature survey, MALDI-MSI and DESI-MSI are the most commonly applied mass imaging methods for TCM investigations. DESI is generally utilized for the analysis of polar compounds, partially because the conventional spray solvent used in DESI contains large proportion of water, which hinders the solubility of non-polar organic molecules in the spray solvents [96]. In order to extend DESI to a wider range of compounds, as well as achieve lower detection limits of different classes of chemicals, the composition of spray solvents is optimized. For instance, non-aqueous solvents have been developed to achieve the detection of hydrophobic compounds [97]. In 2013, a new ternary solvent system was designed by Janfelt's group, making it possible to image non-polar metabolites in the leaves and petals of *Hypericum perforatum* using DESI [98]. To conclude, the detection sensitivity of DESI is related to the composition of spray solvents. As for MALDI analysis, the detection sensitivity is greatly influenced by the selection of matrices. MALDI has been applied for the detection and imaging of various kinds of chemicals, including polar and non-polar compounds [81]. Though MALDI holds a wide coverage of detectable compounds, it is still necessary to adjust the matrix according to target compounds. The selection of an appropriate matrix not only improves the detection sensitivity, but also eliminates the interference coming from matrix complexes [93].

The application of MSI enables in situ analysis of various endogenous molecules present in different organs of plants, including root, stem, leaf, flower seed and fruit. Samples collected from different parts of medicinal plants hold different physical properties, thus asking for different treatment of sample tissues.

For soft tissues from leaves or flowers, an indirect analytical method is usually adopted to avoid the displacement of chemicals. By pressing the plant samples onto flat industrial materials under manual or mechanical forces, metabolites in original plant tissues are transferred to the flat surface, which is the so-called “imprint”. Imprints of plant tissues could be made on different materials, such as polymer membranes [99] and silica gel plates [100]. The obtained imprint is then analyzed with MSI, which indirectly reflects how compounds are distributed in original sample tissues. It has been proved that indirect MSI method not only maintains the spatial information of in situ metabolites, but also brings higher reproducibility of detected signals [101].

Different from leaves and petals, the treatment of rigid materials such as roots and stems usually goes as following. First, raw materials are cut into certain length using blades. Then, these short pieces are flash-frozen under extremely cold temperature. Sometimes the plant materials will be embedded in different media to guarantee the integrity of tissues. The typical embedding compounds are sodium carboxymethyl cellulose [102], gelatin [103] and agarose [104]. Afterwards, the plant materials are sectioned into thin slices using a cryomicrotome [105]. When it comes to TCM materials extremely hard, sectioning methods other than cryo-sectioning could also be adopted. For example, an investigation chose a dicer and a shaver to section the dried root of turmeric into thin slices [106]. Next, the obtained slices could be directly thaw-mounted onto indium-tin oxide coated glass slides [107], or be attached to the glass slides using a double-sided adhesive tape [108].

After tissue sections or sample imprints are obtained, optical images of the tissue samples should be captured prior to MSI analysis. By overlaying optical image with ion images, localization sites of secondary metabolites in plant tissues become readily evident.

It is worth noting that although the primary microscopic optical image captured with MSI instruments is sufficient to recognize the outline of tissues and define the regions of interest, the spatial resolution of MSI is comparatively low compared to traditional histological methods [96]. A comprehensive incorporation of MSI with classic histology and pathology is adopted to generate more precise and correct understanding of MSI results [109]. Two approaches have been come up with to combine histology with MSI data: performing MSI analysis and histological staining on successive sections or staining the analyzed samples after non-destructive MSI measurement [110]. Optical images of plant tissues could also be additionally acquired with a conventional microscope. For example, when performing MALDI-MSI of gallotannins and monoterpene glucosides in the roots of *Paeonia Lactiflora* [111], optical images were captured using a microscope before the application of matrix. In the investigation on alkaloids in *Datura leichhardtii* seed, light and scanning electron microscopy (SEM) micrographs were obtained to assist with the MSI results [112].

With regard to MALDI-MSI analysis, MSI analysis is carried out after matrix application. The selection of a suitable matrix, together with the deposition method of the matrix, contributes to the detection sensitivity, spatial resolution and mass range of MSI results [113, 114]. Numerous secondary metabolites in TCM plants fall into the category of low molecular weight compounds, the MS signals of which could be interfered by the background signals from classic small organic matrices such as 2,5-dihydroxybenzoic acid (DHB), *α*-cyano-4-hydroxycinnamic acid (CHCA), and 9-aminoacridine (9-AA) [115]. These classic matrices have been commonly applied in TCM analysis, as can be seen in Table 1. Up to now, several strategies are designed to overcome the drawbacks of classic small organic matrices, including on-tissue chemical derivatization of low molecular weight compounds, modification of classic matrices, and the search for alternative matrices with high molecular weight [116]. The novel strategies were proved to be practical in phytochemical investigations, which provides useful information for further improvement of TCM studies using MALDI-MSI [117, 118]. Apart from matrix application, instrumental parameters such as laser intensity and the diameter of laser spots are also important factors influencing the sensitivity and spatial resolution of MALDI detection. As a result, the optimization of these parameters is necessary in the whole analysis process.

**Table 1:** Different matrices applied in MALDI-MSI

Year	Species	Tissue type	Matrix	Refs.
2014	*Glycyrrhiza glabra*	Rhizome	DHB	[105]
2014	*Podophyllum hexandrum* *Podophyllum peltatum*	Root, Rhizome	DHB	[138]
2014	*Cannabis sativa*	Leaf	CHCA	[144]
2015	*Hypericum olympicum* *Hypericum perforatum* *Hypericum patulum*	Leaf	CHCA	[145]
2016	*Panax ginseng*	Root	DHB	[107]
2016	*Paeonia lactiflora*	Root	DHB	[111]
2016	*Panax ginseng* *Panax quinquefolius* *Panax notoginseng*	Root	DHB, CHCA, 9-AA	[137]
2016	*Ginkgo biloba*	Leaf	DHB	[141]
2017	*Tripterygium wilfordii*	Root	DHB	[104]
2018	*Ginkgo biloba*	Leaf	DHB, 9-AA	[142]
2019	*Curcuma longa*	Root	DHB, 9-AA	[106]
2019	*Aquilaria sinensis*	Stem	DHB	[26]
2020	*Ligustrum lucidum*	Fruit	9-AA	[103]
2020	*Salvia miltiorrhiza*	Root, stem, leaf	DHB	[102]
2021	*Fallopia multiflora* *Fallopia multiflora* var. *angulata*	Root	DHB, NEDC	[136]

As for DESI-MSI analysis, the imprints of sample or newly cut materials can be analyzed with MSI instrument right after an optical image is captured. Parameters responsible for spectra quality of DESI are nebulizing gas pressure, solvent flow rate, capillary voltage and geometry of the ion source [119, 120]. As listed in Table 2, different spray solvents are adopted during DESI-MSI experiments, there have been attempts to optimize the composition of spray solvents as well.

**Table 2:** Different spray solvents applied in DESI-MSI

Year	Species	Tissue type	Spray solvents	Refs.
2011	*Datura stramonium*	Leaf	Methanol-H_2_O (50:50, v/v), containing 1% of formic acid	[112]
2011	*Hypericum perforatum*	Leaf	Methanol-H_2_O (50:50, v/v), containing 1% of ammonia	[101]
2013	*Hypericum perforatum*	Leaf, Petal	Chloroform-acetonitrile-H_2_O (1: 1: 0.04, v/v/v)	[98]
2016	*Areca catechu*	Seed	Methanol	[108]
2019	*Mentha piperita*	Leaf	Methanol	[99]
2019	*Rauvolfia serpentine*	Leaf, Fruit, Root	Methanol	[100]

Under the optimum conditions, MSI data are generated from each coordinate of the sample. Then, massive number of profiles are reconstructed to form ion images of desired compounds [26, 102]. The results can be further processed by mathematical methods to get a deeper understanding of MSI data. To carry out in-depth analysis of MSI data, lots of software packages have been developed over the years. Functions of these software packages includes preprocessing of mass spectrometry data, reconstruction of mass images, and multivariate analysis based on mass images [121]. Typical commercial software tools such as FlexImaging (Bruker), High Definition Imaging (Waters), and ImageReveal (Shimadzu), and ImageQuest (Thermo Scientific) are user-friendly. In comparison, open source software tools like OmniSpect [122], Cardinal [123], MITICS [124] and MSiReader [125] are usually executed under MATLAB or R platforms, which makes users without programming background find it difficult to operate [126]. There are also freeware software tools for data visualization, such as msiQuant [127] and OpenMSI [128].

**Fig. 4:**
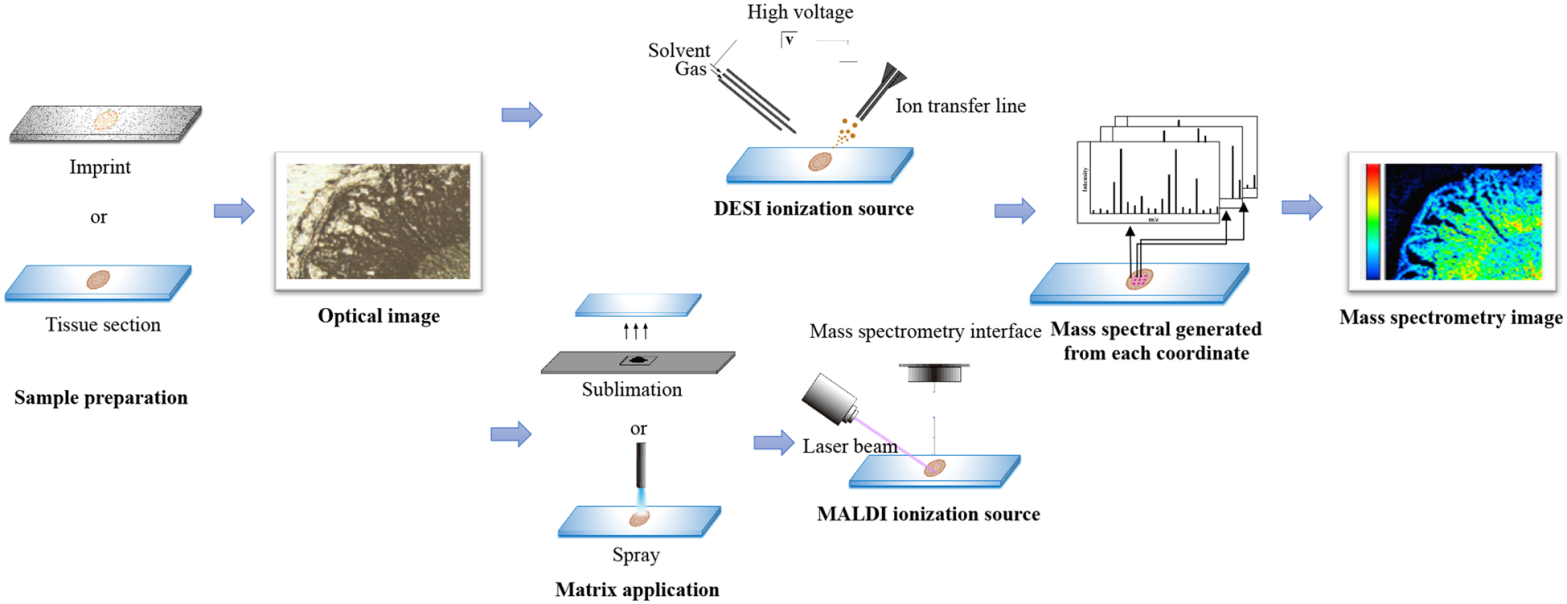
General procedure of TCM investigations using MSI

**Fig. 5:**
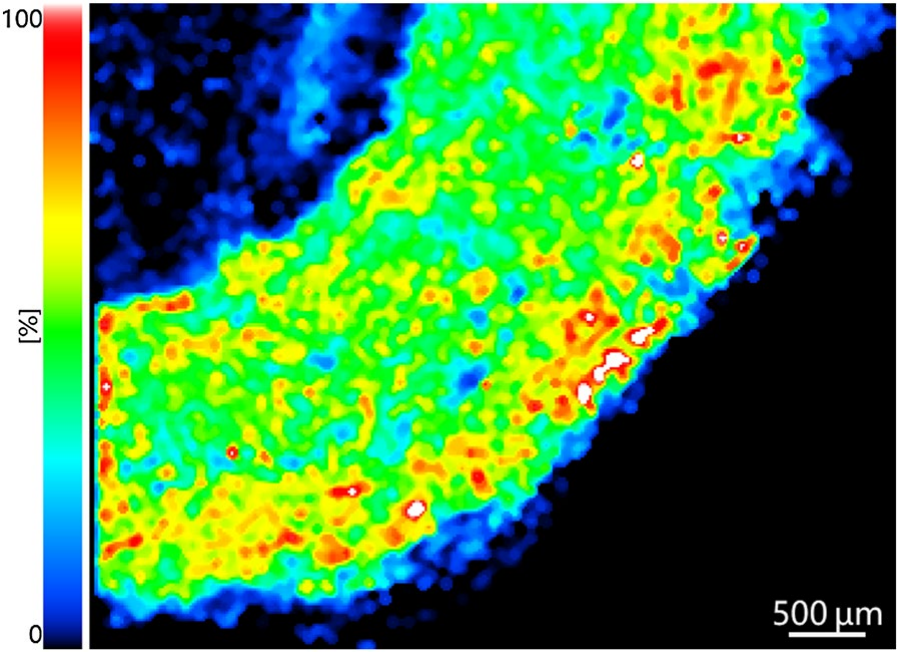
Mass image of the potassium adduct of wogonoside (*m/z* 499.0637) in Scutellariae Radix

The general workflow of MSI in TCM analysis could be seen from Fig. 4. A typical mass image obtained by the authors is shown in Fig. 5, which intuitively demonstrates the distribution of wogonoside in the cross section of Scutellariae Radix. According to the color bar, red color represents high signal intensity, while black color stands for low signal intensity. As a result, it could be readily figured out that wogonoside accumulates in the phloem and xylem, showing a more intense signal in the phloem region.

### Investigations of Traditional Chinese Medicines using MSI

#### Visualization of phytochemicals using MSI

Phytochemicals accumulated in different organs of medicinal plants are the material basis of TCM therapy. Since roots and leaves account for large proportions of TCM materials [129], investigations on MSI of these organs are comparatively more than those of the other organs. A brief summarization of current reports is listed in Table 3, presenting a quick browse through MSI application in TCM research. The utilization of MSI techniques, mostly MALDI-MSI and DESI-MSI, allows the spatial visualization of numerous natural compounds in a single run. Among the two techniques, MALDI-MSI is more frequently used for TCM analysis. Generally, detected chemicals could be identified through several ways: comparison with reference standards, verification of MSI data using complementary information generated from LC–MS results of TCM extracts [102], searching and matching with public MS databases such as Human Metabolome Database (HMDB) [130], MzCloud [131], MassBank [132] and Chemspider [133], as well as self-established databases based on previous literatures [107]. Normally, difference between theoretical and measured masses of the chemicals were expected to be smaller than 5 ppm [105, 111]. After the identification, ion images of different compounds are generated, illustrating the in situ distribution of natural components in an intuitive manner. Apart from the information that can be directly obtained from mass images, a deeper understanding of MSI data could be generated using chemometric calculation.

**Table 3:** Summarization of MSI application in TCM research

Medicinal plants	Organs of plants	Target compounds	MSI techniques	Refs.
*Glycyrrhiza glabra*	Rhizome	Flavonoids, Saponins	MALDI	[105]
*Paeonia lactiflora*	Root	Gallotannins	MALDI	[111]
*Scutellaria baicalensis*	Root	Flavonoids	PALDI	[135]
*Tripterygium wilfordii*	Root	Triterpinoids	MALDI	[104]
*Fallopia multiflora* *Fallopia multiflora* var. *angulata*	Root	Stilbene Glucosides, Anthraquinones, Flavonoids	MALDI	[136]
*Curcuma longa*	Root	Polyphenols	MALDI	[106]
*Panax ginseng* *Panax quinquefolius* *Panax notoginseng*	Root	Triterpenoid Saponins	MALDI	[137]
*Podophyllum hexandrum*	Root	Alkaloids	MALDI	[138]
*Aquilaria sinensis*	Stem	Chromones	MALDI	[26]
*Mentha piperita*	Leaf	Flavonoids	DESI	[99]
*Datura stramonium*	Leaf	Alkaloids	DESI	[112]
*Morus alba*	Leaf	Flavonoid, Saccharides	MALDI	[140]
*Ginkgo biloba*	Leaf	Flavonoid Glycosides, Biflavonoids	MALDI	[141]
*Cannabis sativa*	Leaf	Phenolic Acids	MALDI	[144]
*Hypericum olympicum* *Hypericum perforatum* *Hypericum patulum*	Leaf, Flower	Fatty Acids, Phloroglucinols, Flavonoids	DESI	[145]
*Ligustrum lucidum*	Fruit	Iridoids, Flavonoids, Phenylethanols, Triterpenoids	MALDI	[103]
*Areca catechu*	Seed	Alkaloids	DESI	[108]
*Datura leichhardtii*	Seed	Alkaloids	LADI	[112]
*Ravuolfia serpentine*	Root, Stem, Fruit, Leaf	Alkaloids	DESI	[100]
*Salvia miltiorrhiza*	Root, Stem, Leaf	Phenolic Acids, Quinones	MALDI	[102]
*Ephedra sinica*	Root, Stem	Alkaloids	DART	[146]

#### Root and rhizome

A previous research explored the distribution patterns of flavonoids and triterpene saponins in Glycyrrhizae Radix et Rhizoma by AP-MALDI-MSI. Free flavonoids such as glabrene, licoagroautone and hispaglabridin B were found to localize in particular at the cork layer of the rhizome, which could be correlated with the plant defense function of flavonoids [134]. Differently, licorice saponins tended to accumulate throughout the rhizome except for the cambium region. Glycyrrhizic acid, one of the important saponins in Glycyrrhizae Radix et Rhizoma, showed a relatively high abundance in phloem and xylem regions [105].

Likewise, gallotannins in Paeoniae Radix Alba were visualized with AP-MALDI-MSI. Gallotannins including pentagalloylglucose, hexagalloylglucose, heptagalloylglucose, octagalloylglucose and nonagalloylglucose were all distributed particularly in cork and xylem regions, suggesting their possible function as a barrier against microorganisms. Moreover, a higher resolution of mass images was achieved by performing a scanning step size of 10 μm within the region of interest, offering detailed morphological and chemical information of Paeoniae Radix Alba [111].

In 2014, two major components in Scutellariae Radix, baicalein and wogonin, were characterized with MSI, both showing higher concentrations at the epidermis of the root [135]. Then in 2016, celastrol and demethylzeylasteral, two important triterpinoids in *Tripterygium wilfordii* Hook. F., were visualized with MALDI-MSI. Interestingly, both celastrol and demethylzeylasteral appeared a preferential localization in the periderm. Since periderm is where suberized cork cells locate, researchers hypothesized that there was a co-localization mode existing between lipophilic metabolites and lipophilic cell wall polymers [104].

Polygoni Multiflori Radix, derived from root tubers of *Fallopia multiflora* (Thunb.) Harald., is a famous herb in TCM. One of the major compounds in Polygoni Multiflori Radix is 2,3,5,4’-tetrahydroxystilbene-2-*O*-*β*-D-glucoside (THSG). Images generated from MALDI-MSI showed that THSG presented high ion intensity in secondary phloem, parenchyma and phloem of anomalous vascular bundles of root tubers. Additionally, the untargeted analysis function of MSI allowed the detection of other endogenous metabolites such as quercetin, emodin and physcion. Spatial information of representative natural products might be beneficial for the quality evaluation of prepared slices of Polygoni Multiflori Radix [136].

A recent research succeeded in sectioning Curcumae Longae Rhizoma into 75 ± 5 μm thin slices with a shaver. Sample slices were then visualized under AP-MALDI-MSI. Curcumin, one of the main components in Curcumae Longae Rhizoma, along with its analogs, presented a linear distribution in the longitudinal sections. Whereas bisacurone, another important component in the herb, showed a distribution complementary to curcumin [106].

To take full advantage of the spatial information obtained from MSI data, chemometric calculation methods could be carried out on the basis of MSI results. Principal component analysis (PCA), a multivariate analytical method, is frequently applied to achieve the classification of data collected from different samples.

MALDI-MSI, coupled with PCA, was employed to rapidly differentiate three major commercial species in *Panax* genus, namely, *Panax ginseng*, *Panax quinquefolius*, and *Panax notoginseng*. These species are similar in botanical morphology but present quite different pharmacological functions, thus the discrimination of three species is of great importance. Ginsenosides in roots of the three species were detected with MALDI-MSI. In Ginseng Radix et Rhizoma and Panacis Quinquefolii Radix, ginsenosides tended to store in the cork, while in Notoginseng Radix et Rhizoma, ginsenosides seemed to localize more in the medulla. Afterwards, MSI data collected from ginsenoside-abundant regions were analyzed with PCA. Potential chemical markers which contributed most to the variation between different species were observed from the loading plot. Ions at *m*/*z* of 1147.57, 839.40, 1249.62, 1233.57, 1117.57, 805.40, 985.48, 1203.56 and 1335.61 were selected as chemical markers. The ginsenosides Rb_2_/Rb_3_/Rc/Noto-L (*m*/*z* 1117.57) were observed to exist in Ginseng Radix et Rhizoma and Panacis Quinquefolii Radix, but were undetectable in Notoginseng Radix et Rhizoma. Moreover, ginsenosides Ra_1_/Ra_2_ (*m*/*z* 1249.62) and mRa_1_/mRa_2_ (*m*/*z* 1335.61) showed existence in Ginseng Radix et Rhizoma, but were absent in the other two species. As a result, these chemicals could serve as ideal markers for distinguishing three ginseng species [137].

Similarly, the combination of MALDI-MSI and PCA was adopted to differentiate Ginseng Radix et Rhizoma at the age of 2, 4 and 6 years. In this investigation, a high concentration of ginsenosides were distributed in the cork region of Ginseng Radix et Rhizoma. Cork regions, containing the most abundant ginsenosides in root tissues, were analyzed with PCA. In the score plot generated from PCA, signals collected from the roots of different age clustered into three different groups, indicating distinct difference between the samples. Peaks at *m*/*z* 1127.5 (Ginsenoside Rb_2_/Rc) and *m*/*z* 1147.5 (Ginsenoside Rb_1_) had large intensity variation between the groups. By calculating the intensity ratio of these two peaks (I_1117_/I_1147_), researchers found that 6-year-old ginseng hold the ratio at about 2.45, while the ratio of 4-year-old and 2-year-old ginseng was 0.74 and 2.23, respectively [107].

In another investigation, PCA method was used to classify different cell populations in Paeoniae Radix Alba. Regions of interest were selected within different parts of the root section, then these confined areas were evaluated with PCA. Afterwards, ions contributing to the variance between different botanical structures were sorted out to help differentiate different cell types [111].

As is known, gene duplication gives rise to metabolite diversity in plant tissues. The underlying connection between gene expression and metabolite localization could be a powerful tool to illustrate biosynthetic pathways of natural products. Magnoflorine is a pharmaceutically important alkaloid existing in *Podophyllum* species, the biosynthetic pathway of which affords various aporphine alkaloids. One essential step of this pathway is to convert dopamine and 4-hydroxy-phenylacetaldehyde into norcoclaurine with the existence of norcoclaurine synthase. MALDI-MSI results showed that magnoflorine in the rhizomes mainly localized in the pith and epidermal cells. However, qPCR of dissected tissues rhizome samples suggested that the highest expression levels of norcoclaurine synthase appeared in the xylem region. Such findings indicated that biosynthetic process of magnoflorine occurred in xylem cells at first, then the synthesized metabolite was transported to the rhizome pith and epidermis. Spatial distribution of biosynthesized products helped to understand plant metabolism at a new level [138]. In the similar way, another investigation tried to link the distribution patterns of ginsenosides with their expression patterns of genes within Ginseng Radix et Rhizoma, which might contribute to the commercial production of ginsenosides [139].

#### Stem

In 2019, an investigation combined MALDI-MSI with mass spectral molecular networking, and uncovered novel natural products in Aquilariae Lignum Resinatum. A considerable number of natural products were visualized with MALDI-MSI, among which at least 36 compounds were observed to specifically localized in the resinous heartwood region of *Aquilaria sinensis*, including 14 unidentified compounds. Next, the extraction of stems was analyzed via high-resolution liquid chromatography-tandem mass spectrometry (LC–MS/MS). Profiles generated from LC–MS/MS were then processed through Global Natural Products Society Molecular Networking to establish a MS/MS molecular network. In molecular network, chemicals having similar mass spectral profiles tended to cluster together. Consequently, novel chemicals could be putatively identified according to their adjacent knowns in the molecular network. Based on MSI results, components that distinctively existed in resinous regions were marked out in the molecular network. Interestingly, most of the chemicals clustered into three groups, one representing 2-(2-phenylethyl) chromone monomers, and the other two representing 2-(2-phenylethyl) chromone dimers with the bicyclic structure or *O*-linked structure. In the cluster which represented bicyclic dimer family, an unknown compound (*m*/*z* 595.197) next to PEC E1 (*m*/*z* 565.186) was putatively identified as the methoxylated analogue of PEC E1. In conclusion, the combination of MSI and molecular networking shed light on the study of novel natural compounds [26].

#### Leaf

For the analysis of leaves, indirect analysis method is usually adopted. An investigation placed peppermint leaves between two polytetrafluoroethylene membranes to create an imprint. Metabolites in the leaves of *Mentha piperita* L. were indirectly analyzed with DESI-MSI, including many important flavonoids and their derivatives. Naringenin, a flavonoid which serves as the precursor of all other flavonoids biosynthesized in peppermint plants, was found to localize throughout the leaves, but with less accumulation at the central portion of leaves. Other key components associated with naringenin, luteolin and apigenin routes were also imaged, providing new insights into biosynthetic routes of peppermint flavonoids [99].

Similarly, alkaloids in the leaves of *Datura stramonium* L. were imprinted on a porous Teflon surface and visualized with DESI-MSI, among which atropine and scopolamine were found to accumulate in the ribs and veins of leaves, suggesting that these tropane alkaloids were transported within the plant [112].

Direct MSI analysis could also be employed to analyze natural products in leaves of medicinal plants. Small molecules in Mori Folium were directly detected using MALDI-MSI. Flavonoids including astragalin, rutin, and isoquercetin tended to locate near the veins of Mori Folium, which indicated that secretory canals of flavonoids might locate near the leaf veins. In addition, monosaccharide and disaccharide accumulated mostly in the veins, indicating that sugars were translocated through the veins [140].

For the visualization of components in Ginkgo Folium, direct MALDI-MSI was performed to obtain chemical images. The results showed that flavonoid glycosides distributed all over the leaves, with a higher concentration occurring around the leaf vascular strands. Meanwhile, biflavonoids such as amentoflavone, bilobetin and ginkgetin were found to preferentially accumulate at the underside of the leaves, which could be linked to the physiological functions of biflavonoids acting as a fungitoxin and also feeding deterrent [141]. Apart from pharmaceutical components, allergic toxics in Ginkgo Folium could also be characterized. Mass images of ginkgolic acids and cardanols ions illustrated that secretory cavities of Ginkgo Folium might serve as a storage place for such allergic metabolites. Once an herbivore attacks, allergic toxics in the leaves would be soon released to protect the plant [142].

Typical cannabinoids in *Cannabis sativa* L. were mapped out with MSI, among which tetrahydrocannabinolic acid showed a distinct accumulation in glandular trichomes of leaves, suggesting that glandular trichomes were the storage sites of tetrahydrocannabinolic acid [143]. The detection of cannabinoids via MSI was further utilized to determine whether unwanted cannabis was mixed with therapeutic herbs. A random mixture of cannabis and other herbs was scanned using MALDI-MSI, only leaf pieces that have cannabinoid signals were picked out. Subsequently, morphological examination of selected pieces was carried out as a validation. In leaf pieces that were sorted out under the guidance of MSI signals, characteristic cystolith hairs and trichomes of cannabis were detected with morphological observation, proving that MSI could be adopted to quickly search for cannabis in herb mixtures [144].

As a newly developed technique, MSI still asks for further adjustments to fit different analytical demands. As can be seen in Table 2, methanol is a commonly used spray solvent in DESI, meanwhile, other solvents might also be adopted to promote accurate analysis. In a previous research, fatty acids with extreme long chain in the leaves of *Hypericum perforatum* L. were visualized using DESI-MSI. Detection of these low-polarity compounds was achieved thanks to the introduction of a ternary solution containing chloroform, acetonitrile and water. This unique spray solution allowed the direct visualization of long-chain fatty acids in leaf cuticles, including hexacosanoic acid (C_26_:0), octacosanoic acid (C_28_:0), and melissic acid (C_30_:0). By contrast, the application of conventional binary solvents produced less intense and stable signals [98].

When analyzing components locating beneath the cuticle layer of leaves and petals, cuticles need to be removed since it hinders the detection of inner molecules. Leaves could be attached to an adhesive tape and then lightly pressed for a while, afterwards the tape was gently peeled off to remove cuticles and waxes on leaf surface [145]. There are also moderate ways to deal with cuticle and waxes. In another experiment, leaves of *Hypericum perforatum* L. were dipped in chloroform to take away hydrophobic constituents on the surface. Results showed that chloroform treated parts of leaves presented strong imaging intensities of hyperfirin, adhyperfin, hyperforin, and rutin, while non-treated parts showed weaker signals [101].

#### Flower

Similar to leaves, petals are also soft tissues that may prefer an indirect analytical method. Natural products in the flowers of *Hypericum perforatum* L. were imprinted onto a Teflon surface. Then the imprint of petals was analyzed under DESI-MSI. Images showed that hyperforin was mainly distributed in translucent glands while hypericin was more abundant in the dark glands [101].

#### Fruit

Ligustri Lucidi Fructus, the mature fruit of *Ligustrum lucidum* Ait., is often processed before clinical use to strengthen its pharmacological functions. To reveal the holistic chemical transformation associated with wine steaming preparation of Ligustri Lucidi Fructus, PCA was employed to primarily look into the difference between raw materials and processed materials. Chemometric results unveiled 20 processing-associated markers, among which eight major components were selected to be further analyzed with MALDI-MSI in the raw fruits and 4 h-, 8 h- and 12 h-processed fruits. The results revealed that four major markers (10-hydroxyoleoside dimethylester, 8-demethyl-7-ketoliganin, elenolic acid and salidroside) increased after processing, while the other four (neonuezhenide/isomer, verbascoside/isomer, luteoline and nuzhenal A) showed a decreasing trend. In addition, the spatial distribution of each component was mapped out in the longitudinal section of wine-steamed fruits. Take 10-hydroxyoleoside dimethylester for example, this compound was found to accumulate in the epicarp and mesocarp of raw fruits. However, with the extension of processing time, the concentration of 10-hydroxyoleoside dimethylester gradually increased in the endosperm. Hence, the application of MSI made it possible to simultaneously illustrate temporal and spatial transformation of components in processed Ligustri Lucidi Fructus, which would contribute to the understanding of processing mechanisms [103].

#### Seed

Some medicinal plants are cultivated for their seeds. In Traditional Chinese Medicine, areca nut (seed of *Areca catechu*) is used from ancient times. The inner part of areca nut can be divided into brown and white regions according to the color of each structure. Arecoline, the most abundant alkaloid in areca nut, was visualized with DESI-MSI, showing a preferential occurrence in the brown region of the nut [108].

Besides, with the development of ambient ion sources, plant materials could be analyzed with MSI even without sectioning process. Atropine and scopolamine in *Datura leichhardtii* were directly visualized using laser ablation direct analysis in real time imaging (LADI-MSI). Before MSI, the seed was cut into halves with a razor blade. Then the cut seed was deposited on a silicone putty to keep it stable, with the cut face exposed to MSI device. Finally, clinically important alkaloids were found to distribute in the seed coat, endosperm and the visible embryo. Other alkaloids relating to the biosynthesis of scopolamine and atropine were mapped out as well, providing important information for the study of their biosynthetic pathways [112].

#### Multiple medicinal parts

As listed above, there have been a lot of investigations focusing on the distribution of natural products within a specific plant organ. To gain a deeper understanding of the biosynthesis and transportation process of plant metabolites, it is necessary to simultaneously look into the distributions of a compound in a whole plant.

In 2019, monoterpenoid indole alkaloids in *Ravuolfia serpentine* L. were visualized at a whole plant level using DESI-MSI. DESI images revealed that a majority of indole alkaloids were localized in the roots, with less kinds of indole alkaloids localized in the stem, and only a small proportion of alkaloids were stored in the fruits and leaves. The distribution of alkaloids also presented distinct localization pattern within the same organ. For example, tetraphyllicine, raunescine and deserpidine were distributed specially in the epidermis of roots, while mitoridine and ajmalicine were restricted to the pith region. Temporal changes of natural compounds in *Ravuolfia serpentine* L. were also illustrated with DESI-MSI. In fruits collected at different growing stages, alkaloids such as mitoridine, ajmaline and yohimbine presented higher ion intensities at early growing stage compared to mature fruits. These findings could be further employed to explore the relationship between spatial localization of metabolites and their biosynthetic pathways. Moreover, the accumulation variation of alkaloids in different organs were illustrated. Since alkaloids in the roots presented higher concentration and better diversity, roots were thought to be the primary site of indole alkaloid synthesis in *Ravuolfia serpentine* L. However, this research failed to detect the primary precursors of major alkaloids, which might be due to the structural instability of these compounds [100].

Similarly, an investigation was carried out to study the spatial distribution of bioactive components in different organs of *Salvia miltiorrhiza* Bunge. In Salviae Miltiorrhizae Radix et Rhizoma, phenolic acids and tanshinones are the pharmaceutically important components. MALDI-MSI results showed that phenolic acids such as salvianolic acids were mainly distributed in the xylem and cork formation layer of roots. A portion of the salvianolic acids also existed in the medullary cavity of stems and the veins of leaves, suggesting that salvianolic acids might be synthesized in the root and then transported to other organs. Differently, tanshinones were found to exist only in the periderm of roots, which indicated that tanshinones were synthesized and stored in the roots without being transported elsewhere. After that, tissue segmentation-extraction combined with LC–MS was adopted to serve as a verification of the MSI results. Fresh plants were separated into five tissue parts using a scalpel, the separated parts were extracted and analyzed with LC–MS. In LC–MS results, tanshinones showed highest concentration in the extraction of root periderm, which was consistent with the MSI results. For phenolic acids, a slight mismatching appeared between LC–MS results and MSI results, which might be due to the unavoidable migration of water-soluble chemicals during tissue segmentation process. The comparison between MSI analysis and conventional analytical process proved that MSI was a convenient approach to explore the in situ spatial information of chemicals [102].

More recently, DART-MSI was adopted to visualize major ephedra alkaloids in the whole aerial part of *Ephedra sinica*. Stem and branches linked to the stem were cut longitudinally and then fixed on a glass plate. Later on, both the inner surface and outer surface of the dissected herb were analyzed under direct analysis in real time-time of flight-mass spectrometry (DART-TOF–MS). Mass images showed that the contents of four ephedra major alkaloids, ephedrine, pseudoephedrine, methylephedrine and methylpseudoephedrine, were higher in the branches than in the main stem, and presented a decreasing trend towards the end tip of the branches. Though DART-MSI can’t distinguish diastereomers, it still stands out for the capacity to provide macro-scale molecular imaging of medicinal plants [146].

#### Pharmacological investigations of TCM using MSI

An anti-insomnia drug candidate, N6-(4-hydroxybenzyl)-adenosine (NHBA), was isolated from Gastrodiae Rhizoma. Rats were intraperitoneally injected with NHBA in saline plus Tween 80, then the rats were snap-frozen at 10 min, 20 min, 30 min and 60 min after administration. Sagittal whole-body cryosections of rats were analyzed with AFADESI-MSI. Mass images demonstrated that NHBA was mainly distributed into the intestines of rats, with a small amount accumulating in the brain. Next, MSI data were further analyzed with PCA. Six endogenous metabolites showed significant alteration after the administration of NHBA, including *γ*-aminobutyric acid, choline, valine, creatine, glycerophosphocholine and adenosine. MSI results clearly showed that the concentration of *γ*-aminobutyric acid in the brain of rats increased after the administration of NHBA. Since *γ*-aminobutyric acid is an inhibitory neurotransmitter, the increased level of this compound could serve as an explanation for the sedative and hypnotic effects of NHBA. Similarly, the alteration patterns of the other five metabolites could also be employed to better understand the pharmacological mechanisms of NHBA [27].

Apart from isolated natural compounds, pharmacological effects of crude drug extracts can also be illustrated with MSI. In 2019, researchers explored how the extract of Aconiti Lateralis Radix Preparata improved myocardial damage by affecting the levels of small molecules in the heart. Two groups of rats were intragastrically administered with the extract or total alkaloids of Aconiti Lateralis Radix Preparata respectively. Heart tissue sections in differently treated rats were analyzed by MALDI-MSI to evaluate the alteration of responsive metabolites. Compared with myocardial infarction model group, rats treated with the extract showed increased levels of guanosine monophosphate (GMP) and adenosine diphosphate (ADP) in heart tissues, whereas rats treated with total alkaloids showed increased levels of adenosine monophosphate (AMP), GMP, ADP, adenosine triphosphate (ATP) and creatine. Changed levels of responsive metabolites suggested that components of Aconiti Lateralis Radix Preparata brought a strengthened energy supply in cardiomyocytes. After the treatment of extract and total alkaloids, the dysfunctionl changes of phospholipids in model groups were reversed, indicating another possible mechanism of Aconiti Lateralis Radix Preparata as an anti-myocardial infarction drug [28].

In the same manner, the effects of Shenfu Injection on rats with ischemic heart failure were evaluated with MALDI-MSI. Shenfu Injection is an injectable suspension derived from the extraction of two TCM materials, Ginseng Radix et Rhizoma and Aconiti Lateralis Radix Preparata. After the administration of Shenfu Injection, the contents and distributions of endogenous metabolites in rat hearts presented significant changes. For the injected groups, the contents of antioxidant molecules such as taurine and glutathione reduced compared to model group, suggesting a possible link between the levels of antioxidant molecules and the pharmacological mechanisms of Shenfu Injection. Besides, the spatial distribution of energy metabolism-related molecules was found to vary among different groups. For example, adenosine, one of the downstream products of tricarboxylic acid cycle, distributed differently in the infarct and non-infarct zones after the administration of Shenfu Injection [147].

#### Toxicological investigations of TCM using MSI

MSI has also been employed to evaluate the toxicological mechanisms of a drug. Aristolochic acid (AAI) is a toxic natural component existing in a number of TCM related species, the administration of which would induce nephrotoxicity [148]. Kidney sections of rats treated with AAI were analyzed using AFADESI-MSI. Results showed that tens of metabolites related to important metabolic steps significantly changed in AAI treated groups. Furthermore, mass images collected from AAI treated groups clearly revealed that metabolome alterations in renal cortex were more obvious than in the renal medulla, suggesting that renal cortex was more susceptible to AAI exposure than the medulla [29].

#### Quality control of TCM formulas using MSI

In clinical practice, single ingredients of TCM are usually prescribed in the form of classical formulas. DESI-MSI technique was employed to evaluate the quality of Shaoyao Gancao Decoction and Banxia Xiexin Decoction. Shaoyao Gancao Decoction is composed of Glycyrrhizae Radix et Rhizoma and Paeoniae Radix Alba. Crude drugs from different habitats were extracted to obtain 15 batches of lyophilized powder. Afterwards, different batches of lyophilized powder were dissolved in methanol and dotted on filter paper respectively. Then the filter paper was analyzed by a DESI-MSI system. Index components of Shaoyao Gancao Decoction, including paeoniflorin, liquiritin, glycyrrhizic acid and albiflorin, together with 11 other components, were detected from the 15 dots. The components showed different intensity signals in 15 dots, which could be used to support semi-quantitative analysis [149]. In the same way, index components in Banxie Xiexin Decoction were evaluated [150]. Both investigations brought fresh insights into the quality control method for lyophilized powder and dispensing granules of TCM.

## Conclusions

Over the decades has seen the development of different MSI techniques, more and more commercial ion sources are invented to handle different analytical tasks, including a group of newly developed ambient ion sources. The three most commonly applied techniques, MALDI-MSI, DESI-MSI and SIMS-MSI, are capable of providing complementary types of information according to diverse analytical demands [96]. The performance of MSI analysis is under continuous improvement, in order to achieve higher mass resolution or better spatial resolution [98, 138].

MSI has been proved as a powerful tool for in situ analysis of phytochemicals in plant tissues. With little or no sample pretreatment, various natural compounds could be simultaneously detected in a single run, providing the spatial information of chemicals within plant tissues. The progress made in phytochemistry has put forward the application of MSI in TCM analysis. Localization modes of primary or secondary metabolites within medicinal plants give hints to the illustration of physiological functions and biosynthetic mechanisms of major components in TCM. For sure, the specific accumulation features of TCM constituents illustrated with MSI will serve as a guidance for the commercial production as well as laboratory investigations of TCM components.

Not only pharmaceutically important chemicals but also toxic chemicals can be mapped out in mass images. In an earlier investigation, allergic metabolites in ginkgo leaves were visualized with MSI, which might bring new thoughts to the study of drug safety. Apart from toxic endogenous metabolites, exogenous harmful residues including pesticides, heavy metals and aflatoxins, also exist in TCM. Though there is little research specifically focusing on the distribution of exogenous toxics in TCM investigation, there have been quite a number of papers exploring the distribution of environmental toxics in other plants [151, 152], which might serve as a reference for the study of toxicants in TCM.

Data generated from MSI could be further analyzed with other analytical methods to obtain a deeper understanding of TCM components. The combination of MSI and chemometric methods is usually adopted to classify the data. Using PCA, variants having strong contribution to the group classification will be highlighted in score plots, which is useful for the selection of marker components. The combination of MSI and PCA shows high potential for the differentiation between TCM materials having similarity in botanical features. MSI may also act as a powerful tool to look into the holistic transformation of natural products in processed materials, providing support for the study of processing mechanisms in TCM theory. Aside from chemometric methods, MSI results could be connected with gene expression study, molecular networking, as well as other scientific methods to promote a more comprehensive explanation of MSI data.

Based on the previous progress of MSI in preclinical experiments, MSI has been applied to study the pharmacological mechanisms of TCM. In recent years, the effects of several TCM products have been evaluated with MSI, including a single component isolated from Gastrodiae Rhizoma, crude drug extracts of Aconiti Lateralis Radix Preparata, as well as an injectable preparation derived from the extraction of Ginseng Radix et Rhizoma and Aconiti Lateralis Radix Preparata. MSI uncovers how the contents and distributions of endogenous metabolites are changed after TCM drug administration, which supports the investigation of TCM pharmacology in a novel way. Similarly, the toxicological mechanisms of TCM components could also be illustrated using MSI.

Nevertheless, the application of MSI technique in TCM investigations still has many shortcomings. For instance, it remains a challenge to carry out absolute quantitation using MSI due to sample heterogeneity and ionization suppression effects [153]. Though there has been research focusing on semi-quantitative MSI analysis of natural compounds in plant tissues, absolute quantitation is still an unsolved task. Additionally, MSI could not distinguish between chemical isomers [111]. By improving upon intensity reproducibility, resolution power, and quantitative capabilities, it is anticipated that MSI will make great contribution to the modernization process of TCM.

## Data Availability

The datasets used and/or analyzed during the current study are available from the corresponding author upon reasonable request.

## Abbreviations

TCM: Traditional Chinese medicine
MSI: Mass spectrometry imaging
LC: Liquid chromatography
GC: Gas chromatography
GC–MS: Gas chromatography coupled with mass spectrometry
LC–MS: Liquid chromatography coupled with mass spectrometry
MALDI-TOF–MS: Matrix assisted laser desorption ionization time of flight mass spectrometry
MALDI-MS: Matrix-assisted laser desorption ionization mass spectrometry
LMD: Laser microdissection
LC-QTOF-MS: Liquid chromatography-quadrupole/time of flight-mass spectrometry
UHPLC-QTOF-MS: Ultra-high performance liquid chromatography quadrupole/time of flight-mass spectrometry
MALDI: Matrix-assisted laser desorption ionization
DESI: Desorption electrospray ionization
SIMS: Secondary ion mass spectrometry
LESA: Liquid extraction surface analysis
DART: Direct analysis in real-time
LAESI: Laser ablation electrospray ionization
TOF–SIMS: Time of flight-secondary ion mass spectrometry
AFADESI: Air flow assisted desorption electrospray ionization
MCAEF: Matrix coating assisted by an electric field
PTFE: Polytetrafluoroethylen
TLC: Thin layer chromatography
AP-MALDI: Atmospheric pressure matrix assisted laser desorption ionization
SEM: Scanning electron microscopy
DHB: 2,5-Dihydroxybenzoic acid
CHCA: *α*-Cyano-4-hydroxycinnamic acid
9-AA: 9-Aminoacridine
NEDC: N-(1-naphthyl) ethylene- diamine dihydrochloride
THSG: 2,3,5,4’-Tetrahydroxystilbene-2-*O*-*β*-D-glucoside
PCA: Principal component analysis
LC–MS/MS: Liquid chromatography-tandem mass spectrometry
LADI-MSI: Laser ablation direct analysis in real time imaging
DART-TOF–MS: Direct analysis in real time-time of flight-mass spectrometry imaging
NHBA: N6-(4-hydroxybenzyl)-adenosine
GMP: Guanosine monophosphate
ADP: Adenosine diphosphate
AMP: Adenosine monophosphate
ATP: Adenosine triphosphate
AAI: Aristolochic acid
